# Identification of Effective and Nonpromiscuous Antidiabetic Drug Molecules from *Penicillium* Species

**DOI:** 10.1155/2022/7040547

**Published:** 2022-06-08

**Authors:** Shahzad Saleem, Shabana Bibi, Qudsia Yousafi, Tehzeem Hassan, Muhammad Saad Khan, Mohammad Mehedi Hasan, Hitesh Chopra, Mahmoud Moustafa, Mohammed Al-Shehri, Mohammad Khalid, Atul Kabra

**Affiliations:** ^1^COMSATS University Islamabad, Sahiwal Campus, Sahiwal, Pakistan; ^2^Department of Biological Sciences, Shifa Tameer-e-Millat University, Islamabad, Pakistan; ^3^Yunnan Herbal Laboratory, College of Ecology and Environmental Sciences, Yunnan University, Kunming 10, Yunnan 650091, China; ^4^Department of Biochemistry and Molecular Biology, Faculty of Life Science, Mawlana Bhashani Science and Technology University, Tangail, Bangladesh; ^5^Chitkara College of Pharmacy, Chitkara University, Rajpura, Punjab, India; ^6^Department of Biology, College of Science, King Khalid University, Abha 9004, Saudi Arabia; ^7^Department of Botany and Microbiology, Faculty of Science, South Valley University, Qena, Egypt; ^8^Department of Pharmacognosy, College of Pharmacy, Prince Sattam Bin Abdulaziz University, Al-Kharj 11942, Saudi Arabia; ^9^University Institute of Pharma Sciences, Chandigarh University, Ghruan, Mohali 140413, Punjab, India

## Abstract

Diabetes mellitus (DM) is a very common metabolic disorder/disease. The deterioration of *β*-cells by autoimmune system is the hallmark of this disease. Thioredoxin-Interacting Protein (TXNIP) is responsible for *β*-cells degradation by T-cells in the pancreas. This protein had been declared a good drug target for controlling DM. Lots of side effects have been reported as a result of long-time consumption of conventional antidiabetic drugs. The development of new and effective drugs with the minimal side effects needs time. TXNIP was selected as a target for Computer-Aided Drug Design. The antidiabetic fungal metabolite compounds were selected from the literature. The compounds were screened for their drug-likeness properties by DruLiTo and DataWarior tools. Twenty-two drug-like fungal compounds were subjected to Quantitative Structure-Activity Relationship (QSAR) analysis by using CheS-Mapper 2.0. The lowest (0.01) activity cliff was found for three compounds: Pinazaphilone A, Pinazaphilone B, and Chermesinone A. The highest value for apol (81.76) was shown by Asperphenamate, while Albonoursin and Sterenin L showed highest score (40.66) for bpol. The lowest value (0.46) for fractional molecular frame (FMF) was calculated for Pinazaphilone A and Pinazaphilone B. TPSA for Pinazaphilone A and Pinazaphilone B was 130.51 Å^2^. log  *P* < 5 was observed for all the twenty-two compounds. Molecular docking of fungal compounds with TXNIP was done by AutoDock Vina. The binding energy for complexes ranged between −9.2 and −4.6 kcal/mol. Four complexes, TXNIP-Pinazaphilone A, TXNIP-Pinazaphilone B, TXNIP-Asperphenamate, and TXNIP-Sterenin L, were selected for MD simulation to find out the best lead molecule. Only one complex, TXNIP-Pinazaphilone B, showed a stable conformation throughout the 80 ns run of MD simulation. Pinazaphilone B derived from the *Penicillium* species fungi was selected as the lead molecule for development of antidiabetic drug having the least side effects.

## 1. Introduction

Diabetes mellitus (DM) is a very common disease related to the metabolic disorders. It is prevalent around the world irrespective of the economic conditions [1]. Type 1 DM is more common among the children from infancy to 14 years of age [2]. Type II DM was found to be more prevalent in adults but it has also been reported in children recently [2]. Diabetes had affected 8.5% of persons aged 18 years and above in 2014. Diabetes had been reported as the direct cause of 1.5 million deaths in 2019, with 48% of deaths occurring before the age of 70 years. The premature mortality due to diabetes was found to be more than 5% during the period from 2010 to 2016, with the highest rate being in poor countries. It has been estimated that more than 629 million people will be affected by this disease by 2049 [3]. Type I DM is induced by deterioration of pancreatic *β*-cell by T-cells of human immunity system [4]. Type II DM is related to insulin resistance and is more common in obese people.

The hallmark of the type 1 and type 2 diabetes is the destruction of pancreatic *β*-cells [5, 6]. The treatment strategies for this disease are focused on enhancing *β*-cells functioning by reducing their apoptosis. Thioredoxin-Interacting Protein (TXNIP) has been identified as an important glucose-induced gene [7]. The role of TXNIP as a *β*-cell destructor has been revealed in recent decades [8]. The overexpression of this protein had been reported in diabetic patients [9]. TXNIP is a promising target that has just emerged in this field of study [10]. A good number of antidiabetic drugs have been developed to reduce the TXNIP level in blood [11]. TXNIP inhibition has been shown to have beneficial effects beyond the *β*-cell including various extra pancreatic tissues [12], such as the cardiovascular system [9], kidney [13], and retina [14]. TXNIP has been identified as a crucial regulator of pancreatic *β*-cell malfunction and death, both of which are important factors in the etiology of type 1 and type 2 diabetes. TXNIP appears to be a promising therapeutic target for diabetes, according to growing evidence based on basic, preclinical, and retrospective epidemiological research [15].

Lifelong diabetes medications have some side effects. The drugs might have side effects due to their off-target activities. Angioedema is a disorder that results in larynx, uvula, and tongue swelling, which leads to breathing problems [16]. This is due to off-target activity of antidiabetic drugs against Angiotensin Converting Enzyme (ACE) [17]. Most of the drugs are associated with skin problems [18]. Scars formation is a very common side effect of antidiabetic drugs [19]. Some of the side effects are allergy-related, especially to insulin intake [20]. Sulfonylurea drugs were found to be involved in hyperexcitation of *β*-cells resulting in their apoptosis [21]. This side effect leads to fatigue and exhaustion in patients. About one-third of cases of allergy are due to insulin itself [22] and the rest are related to the components of insulin formulation, that is, zinc protamine and meta-cresol [23]. In addition to side effects, treatment failure in some was reported in some patients using very promising drugs [24]. Some important therapies with their side effects are as follows. First is biguanides: Metformin is usually the first prescribed medicine by the doctors to treat type II diabetes. This improves body's way to use insulin and lowers blood sugar. It may cause serious lactic acidosis after continues use over long time. Second is sulfonylureas: Glipizide is another important drug which controls pancreas to produce more insulin. The patients feel shaky, dizzy, sweaty, and confused due to drastically lowering blood sugar level. Third is meglitinides: nateglinide also controls pancreas and more insulin is produced. This drug is not retained in the body and is excreted rapidly, but it still has side effects like lowering blood sugar and weight gain. Fourth is thiazolidinediones (TZDs) : Pioglitazone works by boosting the insulin use by body. The water retention in body increases, which causes swelling. Weight gain and increase of bad cholesterol are its side effects. Some life-threating effects are bone fracture heart failure and bladder cancer. Fifth is alpha-glucosidase inhibitors: Acarbose is a drug taken with first loaf of meal which slows down the breakdown of carbohydrates. This affects the metabolism of carbohydrates and digestion; some common problems faced by using this medicine are gases, stomach pain, and diarrhea. Sixth is DPP-4 inhibitors: alogliptin helps the pancreas to make more insulin. But associated problems are stuffy nose, sore throat, and stomach pain. Seventh is insulin therapy, which is the last resort for diabetes treatment. The insulin is injected in the body with the help of syringe or pen injector. This treatment is not free of side effects. Some common insulin therapy issues are cough, mouth dryness, dizziness, headache, and rashes.

The fungi have a very unique metabolism system and produce metabolites with diversified nature [25]. The discovery of penicillin by Alexander Fleming in 1929 has proven a to be breakthrough in the fungal metabolites research [25]. Besides, penicillin (antibacterial), echinocandin B (antifungal), cyclosporine A (immunosuppressive), and lovastatin (cholesterol-lowering) are all fungal originated and marketed pharmaceutical drugs. Furthermore, fungal metabolites have been reported to be used in human diet and health improvement [26].

New drug development is a time-consuming and expensive approach, which might be the reason for scarce research new drug development [27]. The approval of new drug molecules from FDA takes more than fourteen years [28] with an estimated cost of production of more than 800 million dollars [29]. Most of the drugs fail in clinical trials, which waste entire efforts and expenses of initial screening [30]. It is important to curtail the cost of production of new drugs and expedite this process at successful and smooth pace. More than half of these problems can be solved by using Computer Aided Drug Design (CADD) methods [31]. A large number of molecules from different databases can be screened out in terms of their toxicity and drug-likeness properties by using CADD techniques [32]. The side effects cause a serious problem in the lifetime of using life-saving drugs. This problem can also be addressed by computational Quantitative Structure-Activity Relationship (QSAR) analysis of selected drug molecules [33]. These CADD approaches can be used in combination with wet lab methods for rapid development of new and effective drugs with lesser side effects [34].

Miglitol and Acarbose are two commonly used commercial antidiabetic drugs [35]. These drugs were found to be associated with the serious liver injuries and gastrointestinal damage in patients using them regularly [36]. Identification and development of new and safer antidiabetic medications with low toxicity and few side effects are of crucial importance. Secondary metabolites from plants have been extensively studied for their possible antidiabetic activities. Different chemicals produced by fungi might be used as active ingredients in new antidiabetic drug development. In this study, we used CADD approaches for designing antidiabetic drugs, targeting TXNIP, from fungal metabolites. The QSAR analysis was done for predicting the least promiscuous drug molecules to avoid side effects.

## 2. Materials and Methods

### 2.1. Retrieval and Preparation of Ligands

Different fungal metabolites with antidiabetic properties were selected from literature survey. Three-dimensional (3D) structures of selected compounds were downloaded from PubChem and ChEBI in SDF format. The ligand molecules were prepared, for docking, by MGL tools in AutoDock Vina. The roots were detected and torsion count was done. The prepared molecules were saved in PDBQT format.

### 2.2. Retrieval and Preparation of Protein

**Figure 1:**
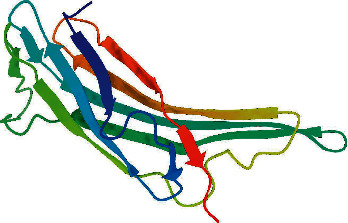
Three-dimensional structure of N-terminal of Thioredoxin-Interacting Protein (TXNIP) (Chain (A) PDB ID : 4GEI) downloaded from Protein Data Bank (PDB).

The Thioredoxin-Interacting Protein (TXNIP) was selected as target protein. The three-dimensional structure of the protein's N-terminal domain was retrieved in PDB format from Protein Data Bank (PDB ID : 4GEI) (Figure 1). The protein was purified by removing water molecules and het-atoms through UCSF Chimera. Polar hydrogen and charges, Gasteiger charge (−9.9935) and Kollman's charge (−211.153), were added by MGL tools of AutoDock Vina. The 3D grid box, as ligand molecule binding site, was adjusted as 39.708 × 33.059 × 4.623 Å. The prepared protein structure was saved in PDBQT format.

### 2.3. Drug-Likeness and Toxicity Analysis of Ligands

The compounds were screened for their drug-likeness by DruLiTo and nontoxicity by DataWarior tools. Mutagenic, tumorigenic, nasty function, irritant, carcinogenic, and reproductive effects were predicted by DataWarrior and the compounds with none of these properties were selected. The Drug-likness parameters like number of hydrogen bond donors/acceptors, log  *P*, and molecular weight were predicted by DruLiTo. The compounds following Lipinski rule of five were selected.

### 2.4. Binding Affinity and Molecular Docking

Molecular docking of N-terminal domain of TXNIP was done with selected (nontoxic and drug-like) fungal compounds (ligands). Protein-ligand blind docking was done using AutoDock Vina tool. The PDBQT files of protein and ligands were used in this process. The results were generated in the form of out.pdbqt file. The out.pdbqt and protein complexes were generated by using PyMOL software. The complex files were saved in PDB format and visualized by LIGPLOT (2D) and BIOVIA Discovery Studio (3D). The protein-ligand complexes showing lowest binding affinities and good hydrogen bond interactions were selected for further studies.

## 3. Quantitative Structure-Activity Relationship (QSAR) Analysis of Selected Compounds

The compounds that qualified the drug-likness and nontoxicity parameters were further analyzed for their Quantitative Structure-Activity Relationship (QSAR) by using CheS-Mapper 2.0 [36]. Statistical approaches were used to compare the predicted and experimental results of compounds. Different QSAR properties were selected for analyses and groups of compounds were created according to their structural features and physical properties.

## 4. Molecular Dynamics (MD) Simulation

Molecular Dynamics (MD) simulation study is a useful strategy towards the understanding of protein-ligand interactions in detail at the atomic level [37]. The top four docked complexes, in terms of lower binding energies, that is, TXNIP-Pinazaphilone A (CID_122182011; *Penicillium* species), TXNIP-Pinazaphilone B (CID_122182012; *Penicillium* species), TXNIP-Asperphenamate (CID_173952; *Penicillium spathulatum*), and TXNIP-Sterenin L (CID_77461068; *Stereum hirsutum*), were selected for MD simulation to find out the best lead molecule. Molecular Dynamics (MD) simulations were performed, for 80 ns time interval, using the Amber v18 software program. For the addition of hydrogen atoms to the protein-ligand combination, the LeaP module was used. To maintain the system's neutrality, counterions (Na^+^ and Cl^−^) were also introduced. For system solvation, a truncated octahedral box of the TIP3P water model with 15 buffers was utilized. For the treatment of long-range electrostatic interactions, the PME (Particle Mesh Ewald) method was used. For simulation, the ff14SB force field was used [38]. Bonds involving hydrogen atoms were constrained using the SHAKE method [39]. To speed up simulations, the PMEMD of the CUDA version of the PMEMD was used [40]. In the NVT ensemble, the steepest descent approach was used to minimize the solvated system for roughly 20000 steps, followed by heating for up to 400 ps and then equilibration for 200 ps. The manufacturing runs at 298 kelvin with a 2 ps time step in Berendsen's thermostat and barostat under the NPT ensemble. The trajectories were analyzed using the CPPTRAJ program.

## 5. Results

### 5.1. Binding Affinity and Interactions in Molecular Docking

The overall binding affinity among docked complexes ranged from −9.2 to −4.6 kcal/mol (Table 1). The lowest binding affinity (−9.2) was observed for one compound (CID_10068406), but no hydrogen bonds were found, so no interaction of ligands was observed with the protein. Only fifteen ligand molecules showed hydrogen bonding with the Thioredoxin-Interacting Protein (TXNIP). Four ligand-receptor complexes formed one hydrogen bond, six ligand-receptor complexes formed two hydrogen bonds, and three complexes formed three hydrogen bond interactions, whereas only two ligand-receptor complexes formed four hydrogen bonds.

### 5.2. Quantitative Structure-Activity Relationship (QSAR) Analysis of Selected Compounds

Only twenty-two nontoxic and drug-like fungal compounds were selected for QSAR analysis (Table 2). The compounds were clustered in two groups (Table 3). In this study, we selected seven features (Table 4) for QSAR analysis of the selected compounds. Activity cliffs (ACs) are pairs or groups of structurally identical compounds which are functional against the same target but have significant potency variances. The two groups of compounds showed more subgroups for activity cliff. Two compounds in cluster I, CID_54436672 (2-methoxy-4,5-dihydroxybenzaldehyde) and CID_528089 (abscisic acid), showed similar value of 0.02. Three compounds from cluster II, CID_122182012 (Pinazaphilone B), CID_122182011 (Pinazaphilone A), and CID_53355009 (Chermesinone A), showed similar value of 0.01. The sum of the atomic polarizabilities (including implicit hydrogens) is calculated as apol [41] and the sum of the absolute values of the difference between atomic polarizabilities of all bonded atoms in the molecule (including implicit hydrogens) is calculated as bpol. The highest value of 81.73 for apol was recorded for CID_173952 (Asperphenamate). For cluster II, two structurally close compounds, CID_77461068 (Sterenin L) and CID_77461069 (Sterenin M), showed the highest value of 75.71, while their other relative CID_77461067 (Sterenin K) showed slightly lower value, 69.52. The highest bpol in cluster I (38.91) was in the case of CID_173952 (Asperphenamate) followed by 29.73 for ChEBI: 171609 (Albonoursin). In cluster II, an overlapping cluster of 3 structurally related compounds showed the highest values of bpol. Two compounds, CID_77461069 (Albonoursin) and CID_77461068 (Sterenin L), shared the same value, that is, 40.66, and CID_77461067 (Sterenin K) showed a value of 36.29. A cluster of two other compounds, CID_122182011 (Pinazaphilone A) and CID_122182012 (Pinazaphilone B), share similar values (28.57) for bpol. Fractional molecular frame (FMF), the key descriptor utilized in the analysis, is based on Bemis and Murcko's hierarchical molecular classification method [42, 43]. Fractional molecular frame (FMF) was calculated for the compounds in both groups. A similar trend to other descriptors was observed. In cluster I, four compounds, CID_173952 (Asperphenamate), CID_10068406 (Benzomalvin A), CID_64435512 (Benzomalvin B), and CID_7090 (Phenidone), shared the same value, that is, 0.92. Similarly, in cluster II, two structurally related compounds shared the same value of 0.47, while two other compounds, CID_12218211 (Pinazaphilone A) and CID_12218212 (Pinazaphilone B), showed 0.46 FMF value. Total polar surface area (TPSA) showed a trend similar to that in other QSAR descriptors. The three structurally related compounds CID_77461067, CID_77461068, and CID_77461069 showed a similar value of 128.16 Å^2^. Two other similar compounds, CID_122182011 and CID_122182012, showed the same value of 130.51 Å^2^. The value of log  *P* < 5was observed for all the compounds. The number of hydrogen bond acceptors <10 and the number of hydrogen bond donors <5 were recorded. These conditions fulfill the requirements for Lipinski rule of 5.

### 5.3. Molecular Dynamics Simulation

**Figure 2:**
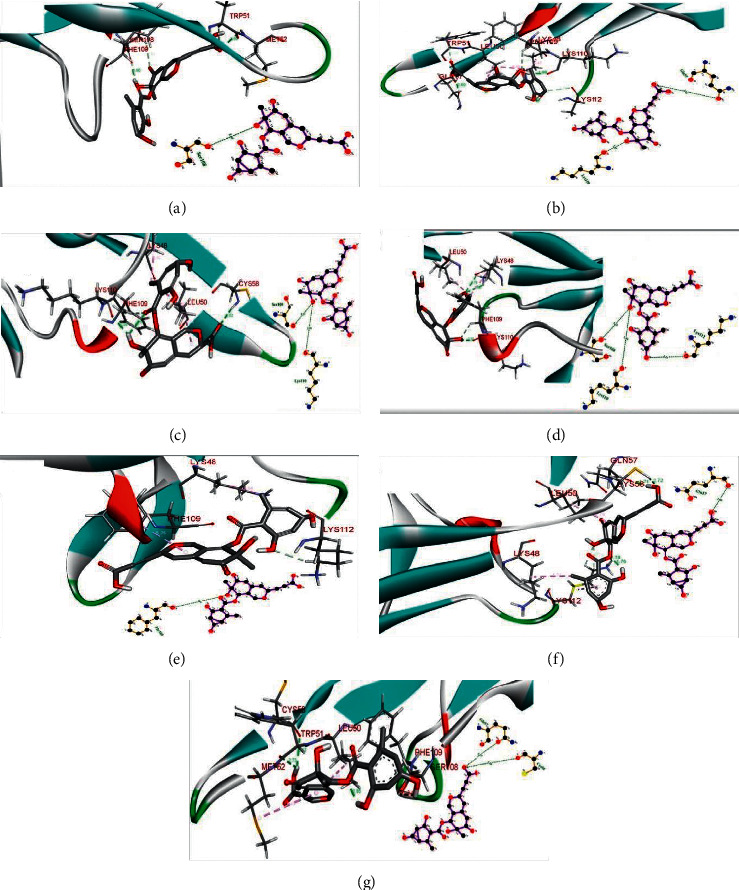
Two-dimensional (2D) and three-dimensional (3D) representations of interactions of Pinazaphilone B-TXNIP complex at different time slots during Molecular Dynamics (MD) simulations. (a) 20 ns, (b) 30 ns, (c) 40 ns, (d) 50 ns, (e) 60 ns, (f) 70 ns, and (g) 80 ns.

The top four selected docked complexes, that is, TXNIP-Pinazaphilone A (CID_122182011; *Penicillium* species), TXNIP-Pinazaphilone B (CID_122182012; *Penicillium* species), TXNIP-Asperphenamate (CID_173952; *Penicillium spathulatum*), and TXNIP-Sterenin L (CID_77461068; *Stereum hirsutum*), were simulated in an explicit water environment for a total 80 ns time period. The complex TXNIP-Pinazaphilone B showed stable behavior throughout the run with four ligand-receptor hydrogen bonds at each time slot (Figures 2(a)–2(g)).

### 5.4. Root Mean Square Deviation (RMSD)

**Figure 3:**
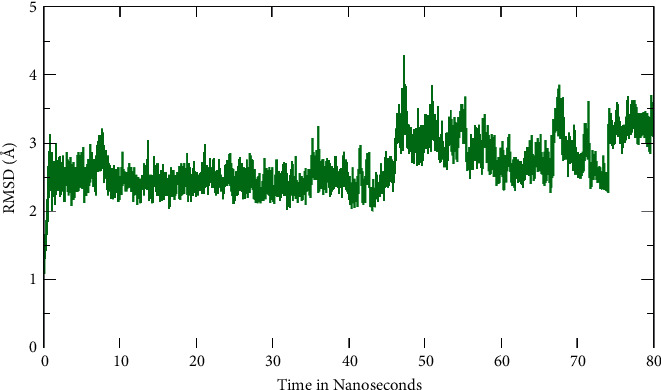
Root Mean Square Deviation (RMSD) fluctuation observed for Pinazaphilone B-TXNIP complex during Molecular Dynamics (MD) simulations run for 80 ns time interval.

The simulation process stability was tested measuring the backbone atoms deviation using Root Mean Square Deviation (RMSD). In general, the RMSD result revealed that the curve showed a progressive increase starting at 1 and oscillating around 2.2 to 3.2 (Figure 3). A peak of 4.3 Å was observed at 49 ns and after that there was a decline, which favors the stability and reliability of the complex.

### 5.5. Radius of Gyration (Rg)

**Figure 4:**
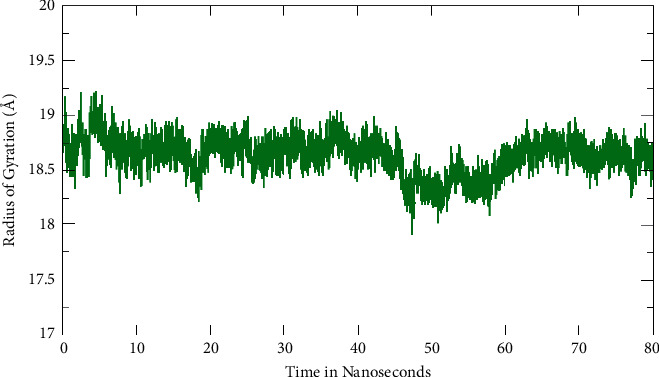
Radius of gyration (Rg) fluctuation observed for Pinazaphilone B-TXNIP complex during Molecular Dynamics (MD) simulations run for 80 ns time interval.

We investigated the complex's overall compactness further using MD simulation and radius of gyration (Rg). The result revealed a very consistent behavior in terms of Rg value below 19.25 Å throughout the MD simulation time (Figure 4).

### 5.6. Root Mean Square Fluctuations (RMSF)

**Figure 5:**
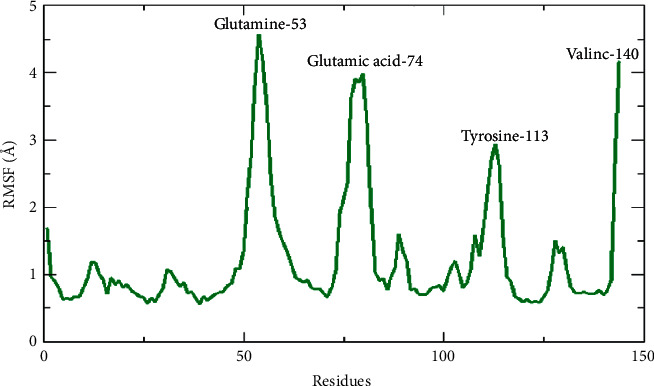
Root Mean Square Fluctuation (RMSF) observed for Pinazaphilone B-TXNIP complex during Molecular Dynamics (MD) simulations run for 80 ns time interval.

We looked into Root Mean Square Fluctuations (RMSF) to better understand the fluctuation of individual residues in the docked complex, which gives us more information about each residue's flexibility. The results of this analysis revealed fluctuation peaks for glutamine 53, glutamic acid 74, tyrosine 113, and valine 140 at 4.5 Å, 3.8 Å, 2.7 Å, and 4.1 A, respectively. The high fluctuation in the docked complex residues might be due to the free movement of the residues, while most of the residues in docked protein showed a steady behavior which might indicate the interaction with the ligand and that is why that region showed a stable behavior with less RMSF curve (Figure 5). The RMSF analysis indicates that the binding of ligand to protein showed a very consistent association.

## 6. Discussion

Diabetes mellitus is a metabolic syndrome which affects millions of people around the world. It is a metabolic disorder of glucose level dysregulation. Different types of drugs are available in the market, among which Miglitol and Acarbose are more commonly prescribed. These drugs hinder the carbohydrate absorption from the gut and can be taken alone or in combination with insulin [44]. As these medicines have to be used for the rest of patients' life, they pose serious side effects in the patients [45]. These associated problems tend to restrict the prescription and uses of certain drugs [36]. This threatening situation of use of conventional antidiabetic drugs leads to an urge for development of some new safer and least promiscuous drugs.

This study was conducted to predict some nontoxic and effective lead molecules, from fungal metabolites, for inhibition of Thioredoxin-Interacting Protein (TXNIP) with minimal side effects. The overexpression of TXNIP causes death of *β*-cells, which is a major cause of diabetes mellitus [46]. Inhibition of this protein had been recommended for targeted antidiabetic drug development. Fungal metabolites have been reported to have many medicinal properties, especially antidiabetic and anticancer nature [47]. These fungal metabolites can be used for synthesizing antidiabetic drugs [48]. The structures of fungal metabolite compounds downloaded from PubChem and ChEBI were screened for their drug-likeness and nontoxic nature. The pharmacokinetic properties, that is, hydrogen bod donors, hydrogen bond acceptors, atomic molar refractivity, number of rotatable bonds, and log  *P* [49], were determined by using Drug Likeness Tool (DruLiTo) 2.1. Lipinski postulated five rules for a molecule to become a drug. He declared that a drug has poor absorption if it has more than 5 hydrogen bond donors, 10 hydrogen bond acceptors, log  *P* greater than 5, and molecular weight above 500 Da. The Lipinski rule of five is popularly followed to define the druggability of the chemical compounds [50]. Twenty-six out of thirty-three compounds passed Lipinski rule of five filter. These compounds were further screened out for their nontoxic nature by submitting to DataWarior tool.

The binding affinities of selected fungal metabolite molecules against TXNIP were investigated using Molecular Docking simulation in this work. Molecular docking is an integral component of Computer Aided Drug Design (CADD) pipeline [51]. This approach can be used to generate protein-ligand interaction complexes with high precision [52] and also for ligand target site identification [41]. The AutoDock Vina tool was used to generate best docked models of protein (TXNIP) and ligands (fungal metabolite molecules) complex. This tool uses scoring functions, in terms of binding affinity, to predict protein-ligand complex interaction strength. Ten different poses, with respective binding energies and RMSD, were generated, which are ranked by scoring functions [53]. The lower the binding energy is, the higher the protein-ligand bonding interaction is. Twenty-two compounds showed good binding affinity between −9.2 and −5.2 kcal/mol.

The selected 22 antidiabetic fungal compounds were also submitted to CheS-Mapper for Quantitative Structure-Activity Relationship (QSAR) Analysis. The pharmacokinetic study of chemical compounds done through QSAR is a very important study in cheminformatics [54]. This approach is used in medicinal chemistry and CADD for lead optimization. In this study, we have studied six descriptors, activity cliffs (ACs), apol, bpol, total polar surface area (TPSA), log  *P*, and fractional molecular frame (FMF), of the selected compounds. Activity cliff of the set of compounds shows their structural closeness but potency might be different [55]. The polarizability is defined as a linear response of electronic charge distribution with respect to an external applied electric field [56]. The study's electronic interaction of chemical compounds is very crucial in the field of medicinal chemistry for proper chemical-protein interaction [57]. Two descriptors of polarizability, apol and bpol, have been calculated for selected compounds in this study. As the antidiabetic drugs are to be taken for the rest of patients' life, the side effects of these medicines are a very serious concern. Different skin disease, heart diseases, and kidney dysfunctions have been reported as antidiabetic-drug-related issues. Promiscuity of a drug can be defined as its ability to interact with multiple proteins and affect their functioning, which results in unrequired pharmacological effects [58]. The promiscuous drugs, if used for a long time, can result in undesired drug metabolism pharmacokinetics [59]. This is an emerging problem in the field of medicinal chemistry and special emphasis has been raised for development of specific and least promiscuous drugs [60]. Three QSAR descriptors, that is, molecular weight, log  *P*, and FMF, are used to describe the promiscuity of a drug molecule [61]. Molecular weight of a drug molecule is very critical for its absorption through the membrane. It is also reported that the promiscuity of the drug molecule is directly proportional to its promiscuity [62]. The molecular weight of the selected lead molecule was 416.40 g/mol, which shows that it has better absorption and has low promiscuity tendency. The quantification of the molecular lipophilicity is generally done by log  *P* value. It is a very critical characteristic of the molecule which should be considered at the time of designing a drug, as higher log  *P* results in higher promiscuity and least transport across biological membrane [57]. The compounds having log  *P* > 5 are more promiscuous, while the least promiscuity was reported in the case of log  *P* > 5 [61]. The selected compound for this study showed log  *P* > 5, while the lead molecule showed log  *P*=0.41, which ensures its least promiscuous nature. The intestinal absorption and blood brain barrier (BBB) penetration of the drug molecule is measured by total polar surface area (TPSA) descriptor [63]. Total polar surface area more than 140 Å^2^ renders least permeability across biological membrane [64]. A molecule with TPSA < 140 Å^2^ has good biological membrane permeability and bioavailability. Most of the compounds in our dataset follow this rule and selected lead molecule (Pinazaphilone B) has TPSA value of 130.51 Å^2^, which reflects its good permeability and bioavailability.

Top four compounds, Pinazaphilone A (CID_122182011), Pinazaphilone B (CID_122182012), Asperphenamate (CID_173952), and Sterenin L (CID_77461068), showing minimum binding energy and good hydrogen bonding with target protein were submitted for Molecular Dynamics (MD) simulation, for lead selection. Molecular simulation protocols are popularly used in CADD pipeline to study detailed structural dynamics at atomic level. The most important use of MD simulation is to test the accuracy of the predicted models in comparison with the experimental results [65]. The standard way to measure the stability of the docked complex is the RMSD of all heavy atoms with respect to the X-ray structure. The RMSD is an important tool that is used to characterize the conformational changes of ligand-protein complexes [66]. The RMSD for the TXNIP-Pinazaphilone B complex showed very little fluctuation in the start and then started to decline; this trend confirms the stability of the complex [67]. The radius of gyration (Rg) is a basic measurement of the overall size of a chain molecule [68]. Each residue's RMSF is simple to calculate across the trajectory, but they can also be translated to temperature factors, which are present for each atom in a PDB file. The Pinazaphilone B-TXNIP complex showed a stable behavior in terms of different MD simulation descriptors.

Pinazaphilone B (CID_122182012) has been identified as an effective inhibitor of TXNIP. This can be qualified as lead molecule for development of effective and least promiscuous antidiabetic drugs. Pinazaphilone B secondary metabolite was extracted from fungus *Penicillium* sp. (Phylum: Ascomycota). It is a well-known fungus in medical history with the discovery of its antibiotic potential [69]. Its adaptable nature increases its presence in diversified environment, that is, air, soil, water, food, and vegetation. This is an economically important genus with its use in medicinal industry and food production and preservation. Some species are environmentally important because of their role as decomposers [70]. Some species are pathogenic in nature and produce a wide range of mycotoxins [71].

## 7. Conclusion

Fungal metabolites can be used in the development of drugs for many diseases. The side effects and toxicity of the medicines can be reduced by using natural compounds like fungal metabolites as active ingredients of drug. The patients of chronic and lifelong diseases are more prone to the harmful side effects of the medicines. Diabetes is one of the most common diseases in which drugs' side effects have been observed in patients, which sometimes lead to life-threating conditions. In this study, fungal metabolites are used to test their efficacy against TXNIP protein. The Computer Aided Drug Designing techniques have been used to develop targeted drug. Further QSAR analysis confirmed the nonpromiscuous nature of the lead molecule. Only one fungal compound, Pinazaphilone B, showed a stable interaction with TXNIP. Pinazaphilone B derived from *Penicillium* fungi was selected as lead molecule in the development of antidiabetic drug having the least side effects. This fungal metabolite can be tested *in vitro* and *in vivo* to confirm efficacy for inhibition of TXNIP. This compound might be used solely or in combination with some commercially available antidiabetic compounds for development of safe, effective, and nonpromiscuous drugs.

## Figures and Tables

**Table 1:** Results of Molecular Docking analysis of fungal metabolite compounds with TXNIP.

S. no.	Compound name	PubChem /ChEBI IDs	Binding interactions	Bond distance (A°)	Binding energy (Kcal/mol)
1	Phenidone (1-phenylpyraactzolidin-3-one)	CID_7090	0	0	−5.2
2	Asperphenamate	CID_173952	N_1_—Lys115 (A): O N_2_—Met57 (A): O	3.10 3.21	−8.3
3	Abscisic acid	CID_5280896	O_4_—Lys115 (A): O O_3_—Met57 (A): O	3.20 3.09	−6.0
4	Benzomalvin B	CID_6443512	0	0	−8.4
5	Benzomalvin A	CID_10068406	0	0	−9.2
6	Sterenin C	CID_24760620	O_2_—Gly119 (A): N O_4_—Gly119 (A): N O_4_—Gly119 (A): O	3.08 3.07 2.80	−7.6
7	Sterenin A	CID_24760622	O_7_—Lys117 (A): O O_5_—Gly19 (A): O	3.30 3.02	−7.5
8	6′-O-Desmethylterphenyllin	CID_53262748	0	0	−7.0
9	Chermesinone A	CID_53355009	O_4_—Gln65: NE2	3.21	−6.4
10	2-Methoxy-4,5-dihydroxybenzaldehyde	CID_54536672	O_1_—Gln81: NE2 O_4_—Lys96: O O_3_—Asn95: OD1	3.13 2.77 2.91	−4.6
11	Sterenin K	CID_77461067	O_8_—Tyr123: O O_7_—Tyr123: N O_6_—Glu144: OE1 O_5_—Phe114: O	2.73 3.17 3.28 3.25	−7.8
12	Sterenin L	CID_77461068	O_8_—Phe114: N O_8_—Gly111: O	2.98 2.94	−7.9
13	Sterenin M	CID_77461069	O_5_—Thr112: O	2.97	−7.9
14	Pinazaphilone A	CID_122182011	O_5_—Met57: N O_5_—Met57: O	2.96 2.88	−8.1
15	Pinazaphilone B	CID_122182012	O_7_—Phe114: O O_7_—Thr112: O O_6_—Thr112: O	3.33 3.24 2.89	−8.1
16	Leucomelone	CID_135457360	O_5_—Phe114: O	2.81	−7.2
17	Ascosalitoxin	ChEBI:2870	0	0	−5.6
18	Cromakalim	ChEBI:3921	N_13_—Phe114: O N_7_—Gly119: O O_22_—Gly119: O O_22_—Gly119: N	3.24 2.80 2.70 3.02	−7.2
19	Hydroxy-abscisic acid (7′-hydroxy-abscisic acid)	ChEBI:20805	O_16_—Met57: O	3.01	−5.4
20	(−)-*cis*-Clavicipitic acid	ChEBI:48269	0	0	−6.5
21	(7R,8R)-*α*-Diversonolic ester	ChEBI:68225	O_6_—Gly119: O O_6_—Gly119: N	3.06 2.86	−6.9
22	Albonoursin	ChEBI:71609	0	0	−6.5

**Table 2:** Fungal metabolite chemical compounds passing through drug-likeness and nontoxicity rules for drug designing against TXNIP.

S. no.	Compound name	Source	PubChem/ChEBI ID	2D structure
1	Phenidone	*Penicillium spathulatum*	CID_7090	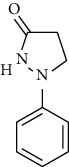
2	Asperphenamate	*Penicillium spathulatum*	CID_173952	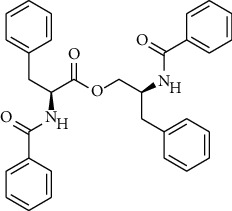
3	Abscisic acid	*Nigrospora oryzae*	CID_5280896	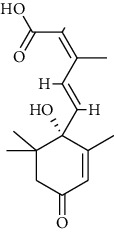
4	Benzomalvin B	*Penicillium spathulatum*	CID_6443512	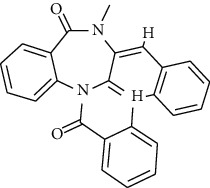
5	Benzomalvin A	*Penicillium spathulatum*	CID_10068406	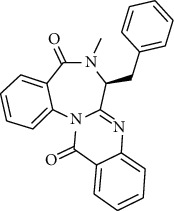
6	Sterenin C	*Stereum hirsutum*	CID_24760620	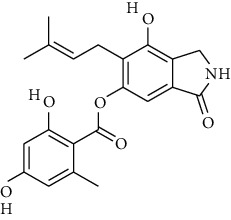
7	Sterenin A	*Stereum hirsutum*	CID_24760622	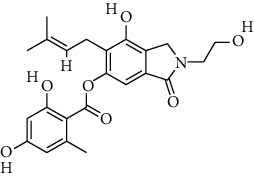
8	6′-O-Desmethylterphenyllin	*Penicillium chermesinum*	CID_53262748	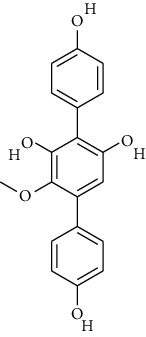
9	Chermesinone A	*Penicillium chermesinum*	CID_53355009	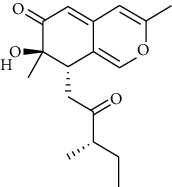
10	2-Methoxy-4,5-dihydroxybenzaldehyde	*Coprinus comatus*	CID_54536672	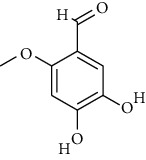
11	Sterenin K	*Stereum hirsutum*	CID_77461067	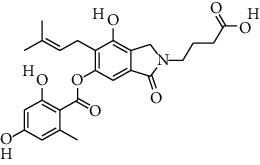
12	Sterenin L	*Stereum hirsutum*	CID_77461068	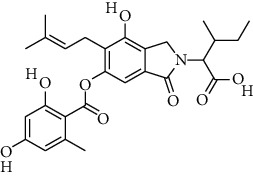
13	Sterenin M	*Stereum hirsutum*	CID_77461069	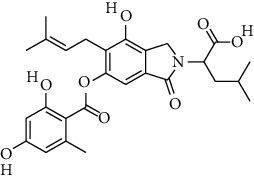
14	Pinazaphilone A	*Penicillium* sp.	CID_122182011	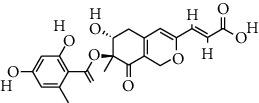
15	Pinazaphilone B	*Penicillium* sp.	CID_122182012	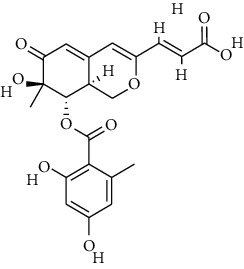
16	Leucomelone	*Sarcodon leucopus*	CID_135457360	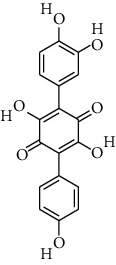
17	Ascosalitoxin	*Penicillium* sp.	ChEBI: 2870 / CID_442951	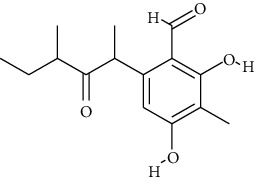
18	Cromakalim	*Penicillium* sp.	ChEBI:3921/ CID_443423	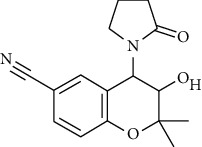
19	Hydroxy-abscisic acid (7′-hydroxy-abscisic acid)	*Nigrospora oryzae*	ChEBI:20805/ CID_11954194	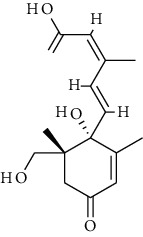
20	(−)-*cis*-Clavicipitic acid	*Penicillium* sp.	ChEBI:482 69 / CID_10061680)	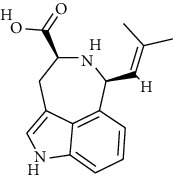
21	(7R,8R)-*α*-Diversonolic ester	*Nigrospora oryzae*	ChEBI:68225/ CID_25058955	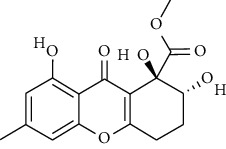
22	Albonoursin	*Penicillium* sp.	ChEBI:71609/ CID_6109346	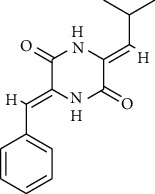

**Table 3:** Clusters of fungal compounds created by CheS-Mapper.

Cluster I		Cluster II	
Compound	Name	Compound	Name
CID_5280896	Abscisic acid	CID_77461068	Sterenin L
CID_53262748	6′-O-Desmethylterphenyllin	CID_77461069	Sterenin M
CID_135457360	Leucomelone	CID_53355009	Chermesinone A
CID_54436672	2-Methoxy-4,5-dihydroxybenzaldehyde	CID_77461067	Sterenin K
CID_173952	Asperphenamate	CID_24760622	Sterenin C
CID_64435515	Benzomalvin B	CID_122182011	Pinazaphilone A
CID_10068406	Benzomalvin A	CID_122182012	Pinazaphilone B
CID_7090	Phenidone	CID_24760620	Sterenin A
ChEBI_71609	Albonoursin	ChEBI_2870	Ascosalitoxin
ChEBI_20805	Hydroxy-abscisic acid (7′-hydroxy-abscisic acid)	ChEBI_68225	(7R,8R)-*α*-Diversonolic ester
ChEBI_3921	Cromakalim	ChEBI_48269	(−)-*cis*-Clavicipitic acid

**Table 4:** QSAR analysis features of fungal compounds.

Name	Compound ID	Mol. wt. (g/mol)	AMR	Activity cliffs	apol	bpol	TPSA	FMF	log *P*	HBA	HBD
Phenidone	CID_7090	162.19	32.34	0.04	25.51	13.21	32.34	0.92	0.24	3	1
Asperphenamate	CID_173952	496.60	84.5	0.04	81.73	38.91	84.5	0.92	4.52	6	2
Benzomalvin A	CID_10068406	381.41	74.6	0.04	59.81	27.97	52.98	0.92	1.92	5	0
Benzomalvin B	CID_6443512	379.41	52.98	0.03	58.48	25.78	52.98	0.92	2.31	5	0
Sterenin A	CID_24760622	383.41	52.98	0.04	63.86	33.14	116.32	0.55	2.52	4	4
Sterenin C	CID_24760620	383.41	116.09	0.05	56.87	27.45	112.51	0.61	2.52	3	4
Sterenin K	CID_77461067	469.50	127.53	0.03	69.52	36.29	128.16	0.52	2.56	5	4
Sterenin L	CID_77461068	497.35	90.15	0.02	75.71	40.66	128.16	0.47	3.79	5	4
Sterenin M	CID_77461069	497.15	63.6	0.02	75.71	40.66	128.16	0.47	4.01	5	4
6′-O-Desmethylterphenyllin	CID_53262748	324.30	66.76	0.03	48.12	19.41	90.15	0.75	1.87	0	4
Leucomelone	CID_135457360	340.12	144.6	0.03	45.3	15.03	135.2	0.72	1.79	4	5
2-Methoxy-4,5-dihydroxybenzaldehyde	CID_54436672	412.13	144.6	0.02	22.62	11.62	66.76	0.53	0.94	1	2
Abscisic acid	CID_5280896	264.32	144.6	0.02	42.94	23.78	74.6	0.32	0.88	4	2
Pinazaphilone B	CID_122182012	416.41	150.59	0.01	57.51	28.57	130.51	0.46	0.41	7	4
Pinazaphilone A	CID_122182011	416.40	150.59	0.01	57.51	28.57	130.51	0.46	0.81	7	4
Chermesinone A	CID_53355009	168.15	135.29	0.01	47.8	27.88	63.60	0.48	1.21	4	1
Albonoursin	ChEBI:71609	256.30	34.14	missing	40.87	29.73	58.2	0.68	3.34	4	2
Hydroxy abscisic acid	ChEBI:20805	280.32	53.33	missing	43.75	23.78	94.83	0.3	0.17	5	3
Cromakalim	ChEBI:3921	286.32	34.14	missing	44.77	25.19	73.56	0.71	0.26	4	1
(7R,8R)-*α*-Diversonolic ester	ChEBI:68225	320.29	17.07	missing	44.44	23.24	113.29	0.61	0.23	5	3
(−)-*cis*-Clavicipitic acid	ChEBI:48269	270.32	52.6	missing	43.97	21.96	78.23	0.65	1.03	3	3
Ascosalitoxin	ChEBI:2870	264.31	34.14	missing	42.94	23.78	74.09	0.32	1.85	2	2

## Data Availability

Data will be available upon request to the corresponding author.
